# Supplementary material for: Pyruvate Dehydrogenase Kinase 1 inhibition mediated oxidative phosphorylation enhancement in cartilage promotes osteoarthritis progression
Source: BMC Musculoskelet Disord. 2023 Jul 20;24:597. doi: 10.1186/s12891-023-06585-6 (PMC10357736; doi:10.1186/s12891-023-06585-6)
Supplement: Supplementary file 1 — Additional file 1. [file 12891_2023_6585_MOESM1_ESM.zip › MF.Gsea.1653627316242/GOMF_ANTIOXIDANT_ACTIVITY.html]

Details for gene set GOMF\_ANTIOXIDANT\_ACTIVITY[GSEA]

|  || Dataset | 55457\_collapsed\_to\_symbols.cls.cls #OA\_versus\_normal.cls.cls #OA\_versus\_normal\_repos |
| Phenotype | cls.cls#OA\_versus\_normal\_repos |
| Upregulated in class | OA |
| GeneSet | GOMF\_ANTIOXIDANT\_ACTIVITY |
| Enrichment Score (ES) | 0.30306643 |
| Normalized Enrichment Score (NES) | 1.3009857 |
| Nominal p-value | 0.099255584 |
| FDR q-value | 0.4093742 |
| FWER p-Value | 1.0 |
Table: GSEA Results Summary

  

Fig 1: Enrichment plot: GOMF\_ANTIOXIDANT\_ACTIVITY      
 Profile of the Running ES Score & Positions of GeneSet Members on the Rank Ordered List

  

| SYMBOL | TITLE | RANK IN GENE LIST | RANK METRIC SCORE | RUNNING ES | CORE ENRICHMENT || 1 | PRDX4 | peroxiredoxin 4 [Source:HGNC Symbol;Acc:HGNC:17169] | 68 | 0.959 | 0.0369 | Yes |
| 2 | PTGS1 | prostaglandin-endoperoxide synthase 1 [Source:HGNC Symbol;Acc:HGNC:9604] | 163 | 0.845 | 0.0668 | Yes |
| 3 | LTC4S | leukotriene C4 synthase [Source:HGNC Symbol;Acc:HGNC:6719] | 223 | 0.804 | 0.0977 | Yes |
| 4 | GSTK1 | glutathione S-transferase kappa 1 [Source:HGNC Symbol;Acc:HGNC:16906] | 490 | 0.662 | 0.1061 | Yes |
| 5 | SOD3 | superoxide dismutase 3 [Source:HGNC Symbol;Acc:HGNC:11181] | 608 | 0.620 | 0.1243 | Yes |
| 6 | MGST3 | microsomal glutathione S-transferase 3 [Source:HGNC Symbol;Acc:HGNC:7064] | 610 | 0.620 | 0.1515 | Yes |
| 7 | SELENOT | selenoprotein T [Source:HGNC Symbol;Acc:HGNC:18136] | 639 | 0.609 | 0.1762 | Yes |
| 8 | GSTZ1 | glutathione S-transferase zeta 1 [Source:HGNC Symbol;Acc:HGNC:4643] | 974 | 0.523 | 0.1732 | Yes |
| 9 | GSTT1 | glutathione S-transferase theta 1 [Source:HGNC Symbol;Acc:HGNC:4641] | 1129 | 0.491 | 0.1828 | Yes |
| 10 | CLIC2 | chloride intracellular channel 2 [Source:HGNC Symbol;Acc:HGNC:2063] | 1214 | 0.473 | 0.1971 | Yes |
| 11 | GSTP1 | glutathione S-transferase pi 1 [Source:HGNC Symbol;Acc:HGNC:4638] | 1260 | 0.465 | 0.2141 | Yes |
| 12 | PARK7 | Parkinsonism associated deglycase [Source:HGNC Symbol;Acc:HGNC:16369] | 1406 | 0.442 | 0.2223 | Yes |
| 13 | SELENOF | selenoprotein F [Source:HGNC Symbol;Acc:HGNC:17705] | 1450 | 0.436 | 0.2381 | Yes |
| 14 | PRDX2 | peroxiredoxin 2 [Source:HGNC Symbol;Acc:HGNC:9353] | 1613 | 0.411 | 0.2436 | Yes |
| 15 | ALOX5AP | arachidonate 5-lipoxygenase activating protein [Source:HGNC Symbol;Acc:HGNC:436] | 1665 | 0.404 | 0.2574 | Yes |
| 16 | CAT | catalase [Source:HGNC Symbol;Acc:HGNC:1516] | 1731 | 0.394 | 0.2697 | Yes |
| 17 | GPX7 | glutathione peroxidase 7 [Source:HGNC Symbol;Acc:HGNC:4559] | 1919 | 0.370 | 0.2714 | Yes |
| 18 | SELENOW | selenoprotein W [Source:HGNC Symbol;Acc:HGNC:10752] | 1944 | 0.368 | 0.2857 | Yes |
| 19 | TXNRD3 | thioredoxin reductase 3 [Source:HGNC Symbol;Acc:HGNC:20667] | 2049 | 0.356 | 0.2933 | Yes |
| 20 | PRDX3 | peroxiredoxin 3 [Source:HGNC Symbol;Acc:HGNC:9354] | 2161 | 0.343 | 0.2998 | Yes |
| 21 | SOD1 | superoxide dismutase 1 [Source:HGNC Symbol;Acc:HGNC:11179] | 2413 | 0.313 | 0.2940 | Yes |
| 22 | MGST2 | microsomal glutathione S-transferase 2 [Source:HGNC Symbol;Acc:HGNC:7063] | 2583 | 0.292 | 0.2937 | Yes |
| 23 | CCS | copper chaperone for superoxide dismutase [Source:HGNC Symbol;Acc:HGNC:1613] | 2727 | 0.278 | 0.2948 | Yes |
| 24 | APOE | apolipoprotein E [Source:HGNC Symbol;Acc:HGNC:613] | 2776 | 0.272 | 0.3031 | Yes |
| 25 | PRDX1 | peroxiredoxin 1 [Source:HGNC Symbol;Acc:HGNC:9352] | 3113 | 0.239 | 0.2874 | No |
| 26 | APOM | apolipoprotein M [Source:HGNC Symbol;Acc:HGNC:13916] | 3415 | 0.210 | 0.2732 | No |
| 27 | GPX5 | glutathione peroxidase 5 [Source:HGNC Symbol;Acc:HGNC:4557] | 3558 | 0.198 | 0.2708 | No |
| 28 | NQO1 | NAD(P)H quinone dehydrogenase 1 [Source:HGNC Symbol;Acc:HGNC:2874] | 3635 | 0.191 | 0.2733 | No |
| 29 | PTGES | prostaglandin E synthase [Source:HGNC Symbol;Acc:HGNC:9599] | 4399 | 0.121 | 0.2192 | No |
| 30 | GSR | glutathione-disulfide reductase [Source:HGNC Symbol;Acc:HGNC:4623] | 4597 | 0.105 | 0.2085 | No |
| 31 | GPX2 | glutathione peroxidase 2 [Source:HGNC Symbol;Acc:HGNC:4554] | 5808 | 0.008 | 0.1146 | No |
| 32 | GSTO1 | glutathione S-transferase omega 1 [Source:HGNC Symbol;Acc:HGNC:13312] | 6275 | -0.025 | 0.0794 | No |
| 33 | TPO | thyroid peroxidase [Source:HGNC Symbol;Acc:HGNC:12015] | 6467 | -0.040 | 0.0663 | No |
| 34 | PRDX6 | peroxiredoxin 6 [Source:HGNC Symbol;Acc:HGNC:16753] | 6783 | -0.061 | 0.0444 | No |
| 35 | APOA4 | apolipoprotein A4 [Source:HGNC Symbol;Acc:HGNC:602] | 6817 | -0.064 | 0.0447 | No |
| 36 | TXNRD1 | thioredoxin reductase 1 [Source:HGNC Symbol;Acc:HGNC:12437] | 6820 | -0.064 | 0.0474 | No |
| 37 | GPX4 | glutathione peroxidase 4 [Source:HGNC Symbol;Acc:HGNC:4556] | 7115 | -0.089 | 0.0284 | No |
| 38 | HP | haptoglobin [Source:HGNC Symbol;Acc:HGNC:5141] | 7360 | -0.107 | 0.0141 | No |
| 39 | NXN | nucleoredoxin [Source:HGNC Symbol;Acc:HGNC:18008] | 7376 | -0.108 | 0.0177 | No |
| 40 | S100A9 | S100 calcium binding protein A9 [Source:HGNC Symbol;Acc:HGNC:10499] | 7509 | -0.119 | 0.0127 | No |
| 41 | GLRX | glutaredoxin [Source:HGNC Symbol;Acc:HGNC:4330] | 7554 | -0.123 | 0.0147 | No |
| 42 | FABP1 | fatty acid binding protein 1 [Source:HGNC Symbol;Acc:HGNC:3555] | 7604 | -0.127 | 0.0165 | No |
| 43 | PXDN | peroxidasin [Source:HGNC Symbol;Acc:HGNC:14966] | 7642 | -0.130 | 0.0193 | No |
| 44 | ALB | albumin [Source:HGNC Symbol;Acc:HGNC:399] | 7684 | -0.133 | 0.0220 | No |
| 45 | IPCEF1 | interaction protein for cytohesin exchange factors 1 [Source:HGNC Symbol;Acc:HGNC:21204] | 7800 | -0.141 | 0.0192 | No |
| 46 | LPO | lactoperoxidase [Source:HGNC Symbol;Acc:HGNC:6678] | 8298 | -0.178 | -0.0116 | No |
| 47 | HBB | hemoglobin subunit beta [Source:HGNC Symbol;Acc:HGNC:4827] | 8775 | -0.215 | -0.0393 | No |
| 48 | HBA1 | hemoglobin subunit alpha 1 [Source:HGNC Symbol;Acc:HGNC:4823] | 8789 | -0.216 | -0.0308 | No |
| 49 | TXN | thioredoxin [Source:HGNC Symbol;Acc:HGNC:12435] | 9487 | -0.273 | -0.0731 | No |
| 50 | MPO | myeloperoxidase [Source:HGNC Symbol;Acc:HGNC:7218] | 9792 | -0.301 | -0.0835 | No |
| 51 | TXNRD2 | thioredoxin reductase 2 [Source:HGNC Symbol;Acc:HGNC:18155] | 9922 | -0.314 | -0.0797 | No |
| 52 | HBQ1 | hemoglobin subunit theta 1 [Source:HGNC Symbol;Acc:HGNC:4833] | 10473 | -0.370 | -0.1063 | No |
| 53 | EPX | eosinophil peroxidase [Source:HGNC Symbol;Acc:HGNC:3423] | 10492 | -0.372 | -0.0913 | No |
| 54 | HBZ | hemoglobin subunit zeta [Source:HGNC Symbol;Acc:HGNC:4835] | 10615 | -0.385 | -0.0839 | No |
| 55 | DUOX2 | dual oxidase 2 [Source:HGNC Symbol;Acc:HGNC:13273] | 10885 | -0.415 | -0.0865 | No |
| 56 | HBE1 | hemoglobin subunit epsilon 1 [Source:HGNC Symbol;Acc:HGNC:4830] | 11153 | -0.451 | -0.0875 | No |
| 57 | UBIAD1 | UbiA prenyltransferase domain containing 1 [Source:HGNC Symbol;Acc:HGNC:30791] | 11299 | -0.471 | -0.0780 | No |
| 58 | HBG1 | hemoglobin subunit gamma 1 [Source:HGNC Symbol;Acc:HGNC:4831] | 11391 | -0.485 | -0.0638 | No |
| 59 | GPX3 | glutathione peroxidase 3 [Source:HGNC Symbol;Acc:HGNC:4555] | 11407 | -0.488 | -0.0434 | No |
| 60 | GSTA1 | glutathione S-transferase alpha 1 [Source:HGNC Symbol;Acc:HGNC:4626] | 11608 | -0.517 | -0.0362 | No |
| 61 | DUOX1 | dual oxidase 1 [Source:HGNC Symbol;Acc:HGNC:3062] | 12336 | -0.687 | -0.0626 | No |
| 62 | HBD | hemoglobin subunit delta [Source:HGNC Symbol;Acc:HGNC:4829] | 12482 | -0.741 | -0.0413 | No |
| 63 | PTGS2 | prostaglandin-endoperoxide synthase 2 [Source:HGNC Symbol;Acc:HGNC:9605] | 12641 | -0.820 | -0.0175 | No |
| 64 | SOD2 | superoxide dismutase 2 [Source:HGNC Symbol;Acc:HGNC:11180] | 12685 | -0.853 | 0.0167 | No |
Table: GSEA details [plain text format]

  

Fig 2: GOMF\_ANTIOXIDANT\_ACTIVITY      
 Blue-Pink O' Gram in the Space of the Analyzed GeneSet

  

Fig 3: GOMF\_ANTIOXIDANT\_ACTIVITY: Random ES distribution      
 Gene set null distribution of ES for **GOMF\_ANTIOXIDANT\_ACTIVITY**
